# Finite element modeling of inter-individual variation in soft tissue mechanical response to localized pressure

**DOI:** 10.1007/s10237-026-02046-w

**Published:** 2026-03-05

**Authors:** Anastasiia Simonova, Aleksei Orlov, Daphne Weihs

**Affiliations:** 1 Faculty of Biomedical Engineering, Technion-Israel Institute of Technology, 3200003 Haifa, Israel; 2 Department of Public Health and Primary Care, Faculty of Medicine and Health Sciences, Ghent University, Ghent, Belgium; 3 Department of Mathematics and Statistics, Faculty of Sciences, Hasselt University, Hasselt, Belgium

**Keywords:** Pressure injury, Finite element biomechanical computer modelling, Layer-specific biomechanical analysis, Stress–strain distribution

## Abstract

Pressure ulcers remain a persistent and serious complication in clinical care, often originating in deep soft tissues before becoming visible on the skin surface and leading to suffering, prolonged hospital stays, and increased healthcare costs. Individual variability in soft tissue composition and mechanical properties plays a critical role in modulating internal stress and strain distributions during prolonged loading. In this study, we used anatomically representative finite element models to investigate inter-individual differences in tissue vulnerability under localized pressure. Two multilayered models, incorporating variations in epidermal, dermal, adipose, and muscular thickness, density, and stiffness, were subjected to clinically relevant pressure magnitudes (2–10 kPa), simulating conditions associated with immobility and device-related compression. Mechanobiological metrics, including effective stress, effective strain, and percentile-based exposure thresholds, were computed to quantify internal tissue load transmission and damage risk. Model outputs revealed that high stress localized in superficial layers, while strain peaked in deeper tissues, especially adipose and muscle. Simulated reductions in tissue stiffness, reflecting age- or disease-related softening, further exacerbated internal loading, increasing stress-exposed tissue volume by up to 1.5 times and strain-exposed volume by up to 1.2 times. These results highlight the biomechanical consequences of anatomical and material variability and support the development of personalized risk assessment tools. The proposed modeling approach contributes to mechanobiology-informed strategies for pressure ulcer prevention in high-risk populations.

## Introduction

Pressure ulcers (PUs), also known as pressure injuries, are localized areas of tissue damage that develop in response to sustained mechanical loading, particularly over bony prominences in patients of all ages in acute, residential, and home care environments (Alvarez-Elizondo et al. 2019; Bååth et al. 2014; Gefen 2020; Jenkins and O’Neal 2010; Lumbley et al. 2014; Reddy et al. 2006; Sezgin et al. 2024; Spilsbury et al. 2007; VanGilder et al. 2017). A substantial subset of pressure injuries originates in deep tissues, such as muscle and adipose layers, where damage may develop before becoming visible at the skin surface (Ankrom et al. 2005; Berlowitz and Brienza 2007; Stekelenburg et al. 2008). Despite widespread clinical efforts, pressure ulcers continue to pose significant risks in acute and long-term care settings, leading to pain, infection, and increased healthcare costs (Kandula 2025; Lyder and Ayello 2008; Mervis and Phillips 2019; Pang et al. 2025). Understanding the mechanical pathways that contribute to internal tissue deformation is thus a central focus of current research on pressure ulcer pathogenesis.

Mechanical factors, including external pressure, shear, and tissue stiffness, play a central role in pressure ulcer formation. However, the body’s internal response to these forces is modulated by anatomical variability across individuals. Key determinants such as tissue thickness, stiffness, and composition influence how mechanical loads propagate from the skin surface into deeper layers (Coleman et al. 2014; Gefen et al. 2022b; Luboz et al. 2014). While prior computational studies have successfully modelled the impact of device-related pressure and thermal effects, relatively few have systematically examined how inter-individual anatomical differences influence the distribution and magnitude of stress and strain across tissue layers under clinically relevant loading conditions. For example, Gefen provides a comprehensive overview of how medical devices that impose pressure or deformation can cause cellular and tissue damage, highlighting the use of computational modeling to investigate device-related pressure effects (Gefen et al. 2022a; Leung et al. 2019; Lustig and Gefen 2022; Orlov and Gefen 2023; Savonnet et al. 2018; Shoham et al. 2017).

Finite element modeling (FEM) provides a powerful tool to simulate tissue mechanics and predict internal load transmission in response to external pressure. This approach allows researchers to test controlled variations in anatomical and mechanical parameters that would be difficult or impossible to isolate experimentally (Levy et al. 2014; Linder-Ganz and Gefen 2004; Verver et al. 2004). In the context of pressure ulcers, FEM has been used to evaluate the mechanical efficacy of support surfaces, interface pressures, and more recently, the effects of microclimate and tissue perfusion (Shaked and Gefen 2013). However, most studies rely on generic anatomical representations and overlook the wide range of inter-individual variability seen in clinical populations (Keenan et al. 2022; Zeevi et al. 2018).

In this study, we present a novel computational investigation into how anatomical and mechanical variability modulates tissue-level mechanical risk under localized pressure. We developed two anatomically distinct finite element models, referred to as Models 1 and 2, based on published data for skin, adipose, and muscle properties (Blaak 2001; Feng et al. 2022; Hattori et al. 1991; Hoffmann et al. 2022; Karla et al. 2016; Lee and Hwang 2002; Levy et al. 2013; Liang and Boppart 2010; Nosslinger et al. 2022; Oltulu et al. 2018; Rabiatul et al. 2021; Sachs et al. 2021; Wang et al. 2023; Zhao et al. 2020). These models differ in tissue thickness, stiffness, and density to reflect real-world variability observed across gender, age, and health status. Both models were subjected to external pressures commonly encountered during device use or immobility (2, 6, 8, and 10 kPa) (Choi and Robinovitch 2011; Derler et al. 2009; Johansson et al. 2002; Joutsen et al. 2024; Taji et al. 2018; Takeshita et al. 2019) and evaluated in terms of effective stress and effective strain across all major soft tissue layers: epidermis, dermis, adipose, and muscle. To better capture clinically meaningful thresholds, we incorporated percentile-based criteria to define tissue exposure to elevated stress and strain. We also explored the effects of tissue softening, mimicking aging or pathological changes, by reducing the stiffness of all soft layers by 10% and 20% (Karla et al. 2016; Lynch et al. 2022; Woessner et al. 2024), respectively. These simulations enabled us to assess how reduced mechanical resistance affects strain propagation and internal tissue vulnerability.

Our work offers several unique contributions. First, by directly comparing two anatomical configurations under identical pressure loads, we isolate the mechanical impact of tissue profile differences, something rarely addressed in the literature. Second, by integrating threshold-based metrics and multi-layer analysis, we provide deeper insights into how and where mechanical damage initiates in soft tissue. Finally, we propose a modeling framework that could be adapted to personalized anatomical data, enabling individualized pressure ulcer risk assessments and targeted preventive care. In doing so, we bridge the gap between mechanical modeling and clinical relevance, offering a toolset that not only explains observed injury patterns but also informs better design of medical devices, repositioning protocols, and support surfaces tailored to individual anatomy. As pressure ulcers remain a persistent and preventable complication in healthcare, advancing personalized biomechanical modeling represents a promising direction for future prevention strategies.

## Methods

### Geometry of the model and the mechanical properties of its components

A square-shaped soft tissue model was constructed with surface-plane dimensions of 120 mm × 120 mm (length × width) exploited by simulating one-quarter of the full model domain, while preserving the mechanical behavior of the complete structure. Two models, referred to as Models 1 and 2, were constructed based on anatomical and mechanical data extracted from previously published literature on the human back region, an anatomical site commonly associated with the development of pressure ulcers. Models 1 and 2 represent average male and female anatomical profiles, respectively, incorporating differences in tissue thickness and mechanical properties reported across adult populations. These gender-based distinctions were introduced for demonstrative and comparative purposes only, to examine the influence of anatomical variability on mechanical outcomes rather than to represent patient-specific conditions.

Each model consisted of five layers: epidermis, dermis, adipose tissue, skeletal muscle, and a rigid underlying bone, consistent with prior multilayer soft tissue modeling approaches (Blaak 2001; Feng et al. 2022; Hattori et al. 1991; Hoffmann et al. 2022; Karla et al. 2016; Lee and Hwang 2002; Levy et al. 2013; Liang and Boppart 2010; Nosslinger et al. 2022; Oltulu et al. 2018; Rabiatul et al. 2021; Sachs et al. 2021; Wang et al. 2023; Zhao et al. 2020). Table 1 summarizes the final tissue thicknesses, Young’s moduli, and Poisson’s ratios used in Models 1 and 2. Epidermal and dermal thicknesses were selected based on histometric and ultrasound measurements of adult skin (Lee and Hwang 2002; Oltulu et al. 2018; Wang et al. 2023). Adipose and muscle thicknesses were derived from population-based anatomical measurements reflecting inter-individual variability in soft tissue composition (Blaak 2001; Hattori et al. 1991; Hoffmann et al. 2022). Elastic moduli for each tissue layer were taken from in vivo and ex vivo mechanical measurements reported in prior studies (Feng et al. 2022; Karla et al. 2016; Liang and Boppart 2010). Where reported values spanned a range, representative mid-range values were selected to define baseline properties, consistent with previous finite element studies of soft tissue mechanics. All soft tissues were assigned a Poisson’s ratio of 0.48, reflecting near-incompressible behavior commonly assumed in biomechanical modeling of soft tissues. Models 1 and 2 differ in both tissue thickness and stiffness to represent inter-individual anatomical variability observed across adult populations. These differences were introduced systematically to isolate their influence on internal stress and strain distributions under identical loading and boundary conditions.

**Table 1:** Soft tissue model components and material properties

Model component	Thickness (mm)		Young’s modulus (kPa)	Poisson’s ratio	Numbers of elements	
	Model 1	Model 2			Model 1	Model 2
Epidermis^a,b,f,p^	0.1	0.07	1500	0.48	40,318	46,901
Dermis^a,f,g,h,p^	1.5	0.84	35	0.48	29,802	20,774
Adipose^d,e,j,m^	4.4	3	2	0.48	64,423	48,869
Muscle^b,k,n^	6.6		21	0.48	98,980 98,980	
Bone^i,o^	5		6480	0.3	37,890 37,890	
Sensor^r^	10		1300	0.45	37,732	

^a^Blaak (2001)

^b^Cui et al. (2022)

^c^Feng et al. (2022)

^d^Hattori et al. (1991)

^e^Hoffmann et al. (2022)

^f^Karla et al. (2016)

^g^Lee and Hwang (2002)

^h^Liang and Boppart (2010)

^i^Linder-Ganz and Gefen (2004)

^j^Luboz et al. (2014)

^k^Lynch et al. (2022)

^l^Nosslinger et al. (2022)

^m^Rabiatul et al. (2021)

^n^Shoham et al. (2017)

^o^Verver et al. (2004)

^p^Wang et al. (2023)

^q^Zeevi et al. (2018)

^r^Zhao et al. (2024)

To represent physiological and pathological variability, additional models were created by applying uniform reductions in tissue stiffness (− 10% and − 20%), consistent with reported age-related and disease-associated softening of skin and underlying soft tissues (Karla et al. 2016; Lynch et al. 2022; Woessner et al. 2024).

**Fig. 1:**
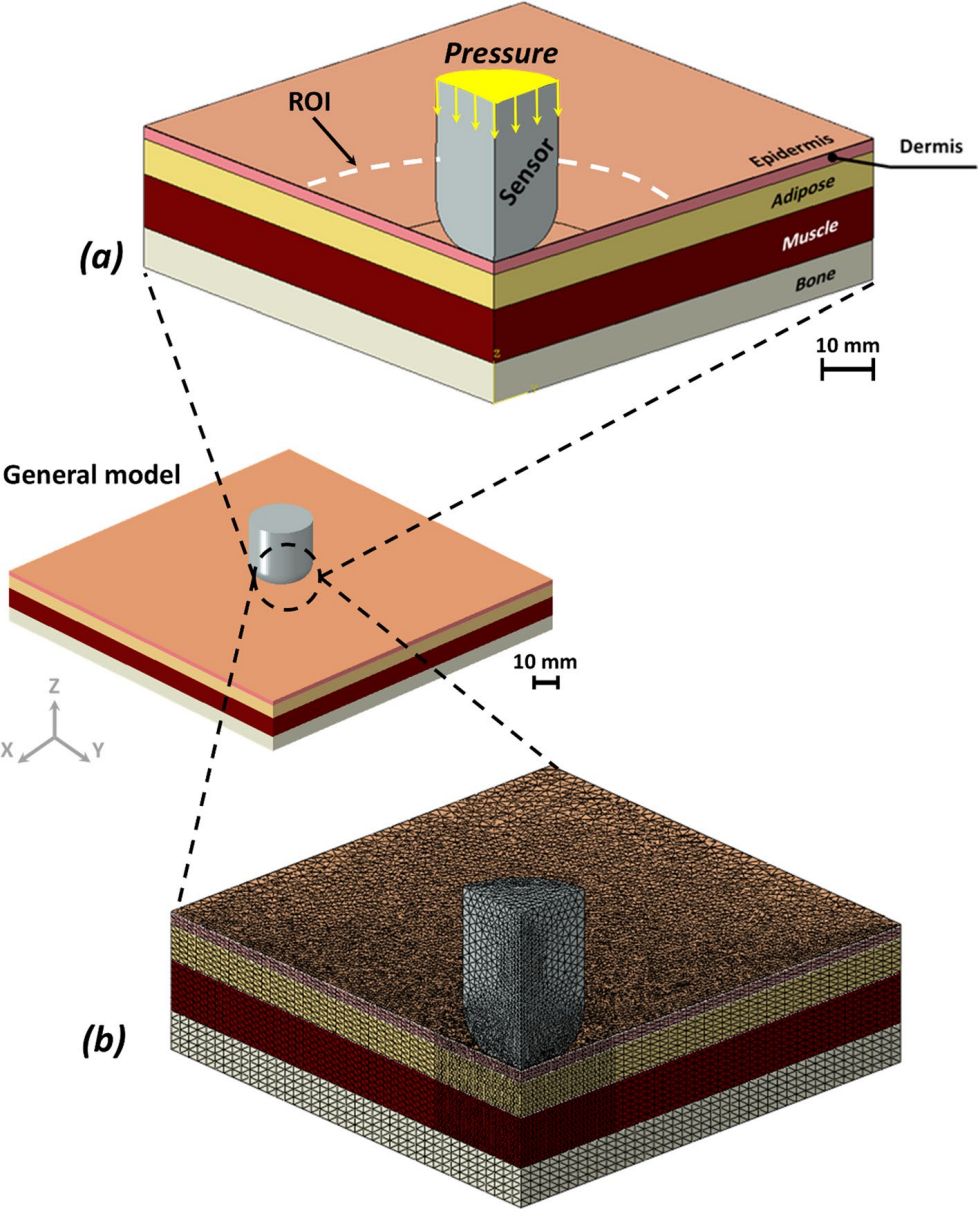
Skin model geometry and finite element modeling under forces applied by round sensor: **a** cross-section of model with its multiple soft tissue layers, location of force-applying sensor, and region of interest (ROI). **b** The mesh of the sensor and soft tissues in its vicinity for finite element modeling; the mesh density was increased towards the middle of the model and deep within the tissue layers. Element counts are reported in Table 1

Localized pressure loading was applied using a circular electrode (sensor) with a diameter of 10 mm, representative of a contact-based device such as a pressure sensor or therapeutic probe (Cui et al. 2022; Zhao et al. 2024). The electrode was positioned in direct contact with the epidermal surface (Fig. 1a).

The region of interest (ROI), defined as a 30 mm diameter zone beneath the electrode extending through the dermis, adipose, and muscle layers, was specified a priori to capture the spatial extent of mechanical loading and to enable consistent comparative analysis across models and loading conditions.

### Boundary and loading conditions in the computational modeling

The contacts between the different tissue layers (epidermis–dermis, dermis–adipose and adipose–muscle) were all set as ‘tie’ (‘no-slip condition’) (Fig. 1a). To replicate in vivo constraints, the basal surface of the bone was fixed in all degrees of freedom, simulating attachment to deeper skeletal structures. Lateral boundaries were constrained against displacement perpendicular to their surface planes to prevent artificial expansion or contraction of the tissue domain. Localized loading was applied using a rigid circular electrode (sensor) in direct contact with the epidermal surface, without mechanical adhesion, allowing free deformation of the skin under compression (Fig. 1a). Contact between the sensor and the epidermal surface was defined as frictionless normal contact, allowing tangential sliding without resistance while transmitting compressive pressure. Uniform normal pressure was applied over the 10 mm diameter electrode area, with four pressure magnitudes (2, 6, 8, and 10 kPa) applied independently in separate simulations to evaluate tissue responses under varying loading conditions (Choi and Robinovitch 2011; Derler et al. 2009; Johansson et al. 2002; Joutsen et al. 2024; Taji et al. 2018; Takeshita et al. 2019). The selected pressure range represents localized interface pressures associated with medical devices, monitoring equipment, and sustained contact during immobility, rather than full body-weight loading. These magnitudes are consistent with experimentally reported skin–device interface pressures back and were therefore chosen to capture local tissue deformation beneath a confined contact area rather than global load-bearing behavior.

Material parameters were not calibrated to a specific experimental dataset, but were selected to ensure internal consistency across tissue layers and models. All soft tissues were modeled using the same constitutive formulation with near-incompressible behavior, and stiffness ranges consistent with published multilayer soft-tissue models. Relative stiffness contrasts between layers were preserved to reflect physiological load-sharing mechanisms, enabling meaningful comparison of mechanical responses between Models 1 and 2.

Boundary conditions were selected to represent a locally constrained tissue region subjected to prolonged external compression. The fixed basal bone surface approximated skeletal support, while symmetry planes and lateral constraints ensured mechanical stability without introducing artificial boundary effects. Although alternative boundary conditions (e.g., frictionless interfaces or compliant supports) could influence absolute stress and strain magnitudes, the consistent application of the selected conditions across all simulations ensures that comparative trends reflect differences in tissue composition and stiffness rather than boundary artifacts.

### Computational simulations, numerical method and outcome measures

Each of the aforementioned soft tissue types excluding bone tissue was assumed to behave as a viscoelastic solid. The hyperelastic component of this viscoelastic behaviour was considered to be Neo-Hookean (Hendriks et al. 2006; Katzengold et al. 2018), with a strain energy density function *W*:

1 $$W= \frac{\mu }{2}\left({I}_{1}-3\right)- \mu \mathrm{ln}J+ \frac{\lambda }{2} {\left(\mathrm{ln}J\right)}^{2},$$

where *λ* and *μ* are Lame's first and second parameters, respectively, *I*_1_ is the first invariant of the right Cauchy–Green deformation tensor and *J* is the determinant of the deformation gradient tensor. The viscous, stress relaxation component of the viscoelastic behaviour of each of the individual tissue layers was represented using a Prony-series:

2 $$G\left(t\right)=1+ \sum_{i=1}^{N}{\gamma }_{i}{e}^{-t/\eta },$$

where *γ*_*i*_, *τ*_*i*_, and *i* = 1, 2,…,*N*, are the tissue-type-specific material constants. For efficiency of the model formulation and computations, we set N = 2 for all tissues, which yields short-term and long-term viscoelastic relaxation time constants for each soft tissue type, *τ*_1_ and *τ*_2_, respectively. The *λ* and *μ*, *τ*_1_ and *τ*_2_, and *γ*_1_ and *γ*_2_ parameter values for each tissue type were selected based on published literature, as detailed in Table 1. Specifically, the bone tissue and sensor were modelled as a homogeneous, linear-elastic, and isotropic material.

The tissue model, as well as the sensor, were meshed using the four-node linear tetrahedral hybrid element (C3D4H) which is particularly effective in managing the incompressibility of biological materials and biomaterials, thereby avoiding undue stiffness in regions of the model. The numbers of elements for each tissue type are further listed in Table 1. The final mesh was selected as an optimal balance between numerical accuracy and computational efficiency; further mesh refinement did not meaningfully change stress or strain distributions or the reported relative trends, but substantially increased computation time. Mesh refinement was highest in the ROI directly beneath the sensor (Fig. 1b), where stress and strain gradients were expected to be most pronounced. The full geometry and mesh were generated using the ABAQUS software suite (ver. 2024, Dassault Systemes Simulia Corp., Johnston, Rhode Island, USA). All simulations were conducted in ABAQUS/Standard (2024) using a nonlinear static implicit formulation with large-deformation kinematics (NLGEOM = YES) and automatic time incrementation (initial increment 0.05, total step time 1.0, minimum increment 1 × 10^−6^, maximum increment 1.0; up to 1000 increments). No artificial numerical stabilization was applied, and default ABAQUS convergence criteria were used. Numerical stability was ensured by bounded incrementation and smooth pressure loading, with no persistent cutbacks observed in the final simulations.

The model was analysed for the von Mises (effective) stresses and effective strains in skin tissue and underlying tissues with a particular focus on the predefined 30 mm diameter ROI located beneath the applied pressure. Results were analyzed layer by layer, epidermis, dermis, adipose tissue, and muscle, to assess how mechanical loads propagated through the different strata of soft tissue.

To further investigate the influence of altered tissue mechanics on biomechanical outcomes, additional model variants were developed by uniformly reducing the Young’s modulus values of all soft tissue layers by 10% and 20% (Karla et al. 2016; Lynch et al. 2022; Woessner et al. 2024). These reduced-stiffness models preserved all other aspects of the baseline simulations, including geometry, boundary conditions, and pressure loading. This parametric extension allowed evaluation of how reductions in tissue stiffness, representative of age- or disease-related degradation, affect the propagation of stress and strain within the skin and underlying tissues.

### Quantitative metrics: averages, thresholds, and AUC calculations

To facilitate comparative analysis of tissue-level mechanical responses across models and loading conditions, several quantitative measures were extracted from the simulation results. For each tissue layer and pressure scenario, average values of von Mises stress and effective strain were computed within the 30 mm diameter ROI, providing a summary measure of overall mechanical loading.

In addition to average values, threshold-based metrics were derived to quantify the spatial extent of high mechanical exposure. Specifically, the 75th percentile values of von Mises stress and effective strain obtained from Model 1 were defined as reference thresholds. These cut-offs were then used to determine the proportion of tissue volume exceeding elevated stress or strain levels, enabling direct comparisons across models, loading magnitudes, and stiffness conditions. The use of the 75th percentile from Model 1 provides a relative high-exposure reference that captures the upper tail of the stress and strain distributions while maintaining consistency across all comparisons. Importantly, this percentile-based threshold does not represent a physiological injury criterion; rather, it serves as a standardized comparative metric when absolute, tissue-specific injury thresholds are uncertain or heterogeneous.

To characterize the cumulative burden of mechanical exposure, area under the curve (AUC) values were calculated from cumulative histograms of stress and strain distributions within each tissue layer. These cumulative distributions describe the fraction of tissue volume exposed to increasing stress or strain magnitudes, and the corresponding AUC values provide an integrated measure of both exposure magnitude and spatial extent.

To quantify the relative extent of high-stress or high-strain exposure, a normalized metric was computed as:

3 $$\text{Tissue Exposure}=\frac{\text{AUC from Threshold}}{\text{AUC Total}}\times 100.$$

This metric represents the percentage of the total tissue-level mechanical load that exceeds the defined threshold, allowing for standardized inter-model and inter-condition comparisons of high-exposure tissue fractions, independent of absolute stress or strain magnitudes.

## Results

**Fig. 2:**
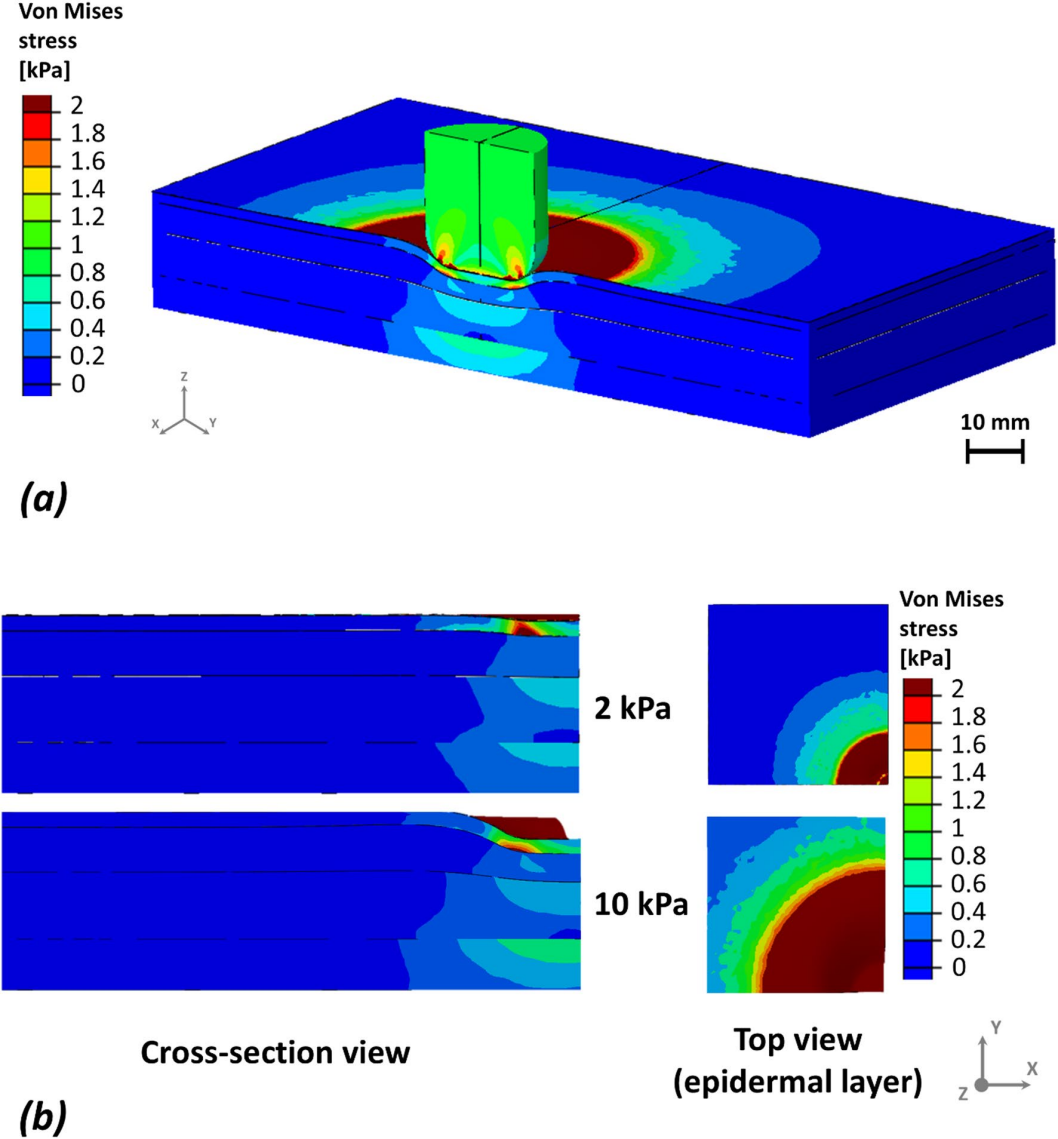
An example of the von Mises (effective) stresses distributions in response to 10 kPa pressure applied by the sensor on Model 1. **a** Stress distribution near the sensor shows effects deep in the tissues. **b** Cross-section and top views, with sensor hidden, demonstrate the stress concentration patterns on the surface of the skin

**Fig. 3:**
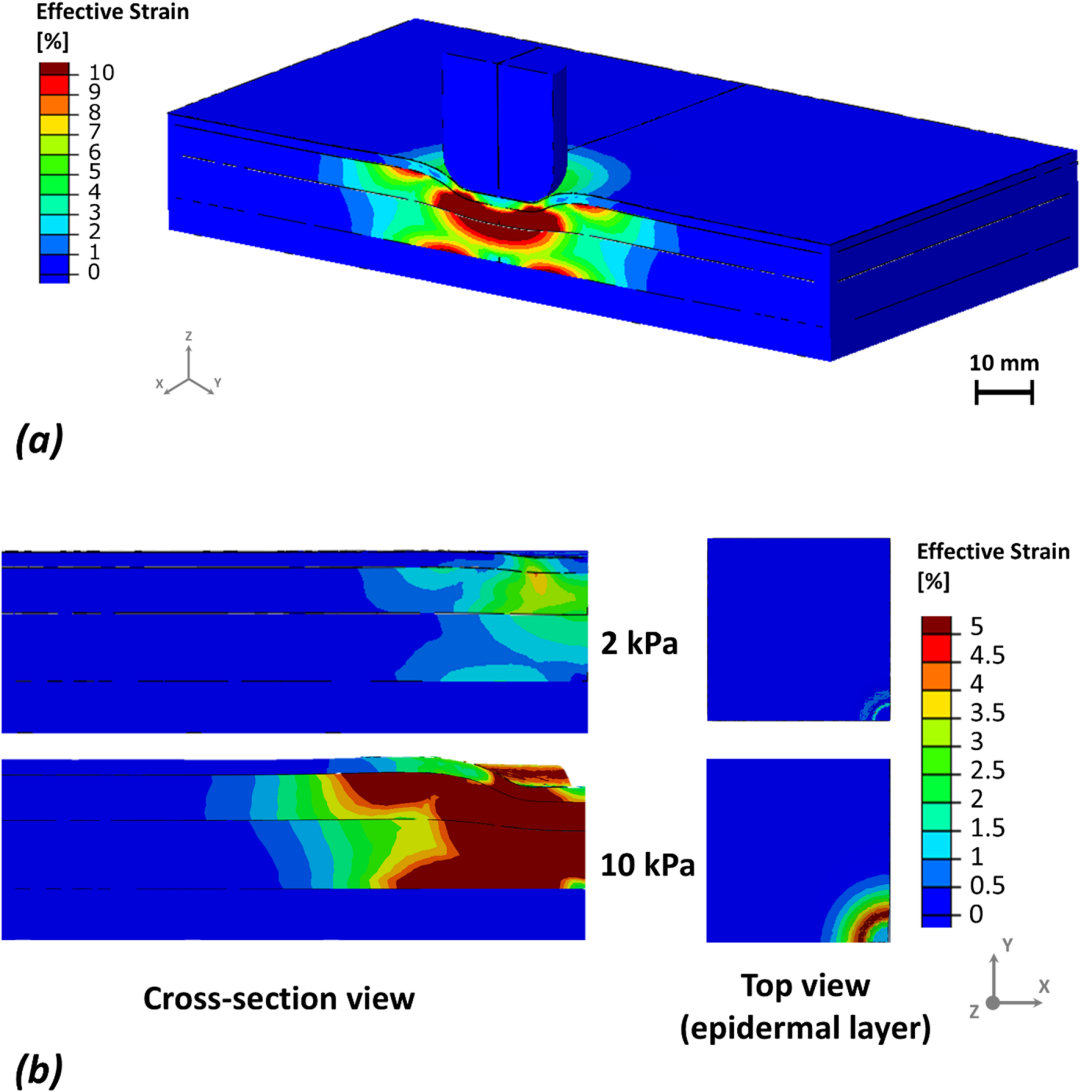
An example of the effective strains distributions in response to 10 kPa pressure applied be the sensor, showing the strain concentrations around the sensor and in the deep tissues (**a**). The sensor was hidden for the cross-section and top views (**b**) to reveal the strain concentration patterns on the surface of the skin

To evaluate the mechanical impact of localized loading, effective stress and effective strain distributions were examined across pressure magnitudes from 2 to 10 kPa (Figs. 2, 3). At 10 kPa, effective stress concentrated predominantly in the epidermis beneath the sensor, with a steep decay with depth. In Model 1, the approximate stress ratio across epidermis, dermis, adipose, and muscle was 47:2.5:1:1.2, indicating strong superficial stress localization (Fig. 2b). A similar depth-dependent trend was observed in Model 2, albeit with relatively greater transmission toward deeper tissues.

At lower loading (2 kPa), stress remained confined to superficial layers, with epidermal stress exceeding deeper tissues by more than an order of magnitude, while adipose and muscle experienced minimal stress (Fig. 2a). In contrast, effective strain exhibited an opposite depth profile, localizing primarily in deeper tissues. At 10 kPa, strain in Model 1 followed an approximate ratio of 1:2:5:1.3 across epidermis, dermis, adipose, and muscle, respectively, peaking in adipose and remaining elevated in muscle (Fig. 3b). Model 2 showed a comparable adipose-dominant pattern but with higher relative muscle strain. Notably, at 2 kPa, strain continued to localize in adipose tissue despite low surface pressure, indicating the onset of deep-tissue deformation even under modest loading.

**Fig. 4:**
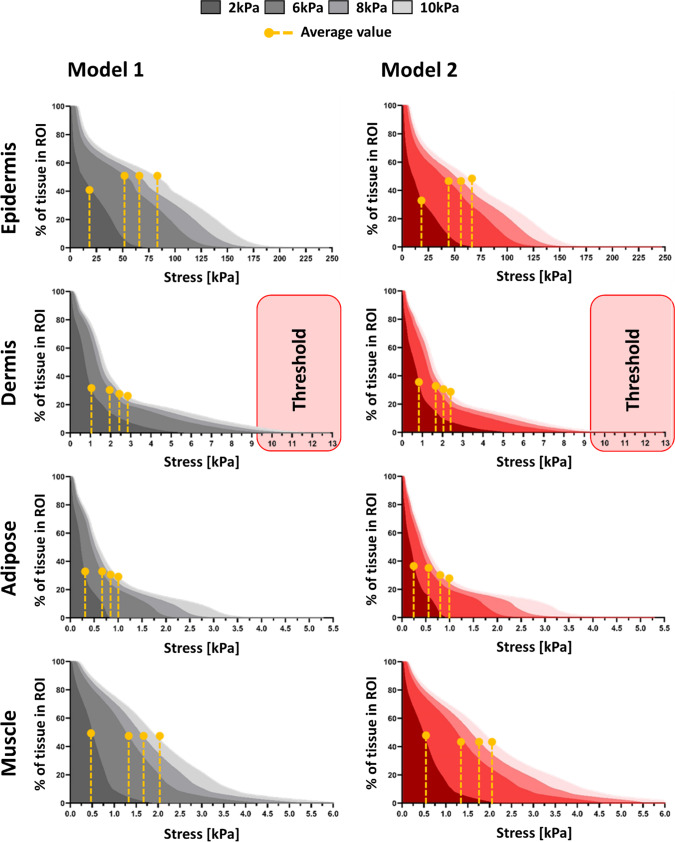
The volumetric soft tissue exposures to von Mises stresses at the ROI of the two studied models (Model 1: left column, Model 2: right column). For each tissue type, the 75th-percentile of the stress domain of the Model 1, i.e. resulting in tissue exposure to highest stress, were defined as the threshold for further analysis and compare the volume of tissue experiencing potentially damaging stress

**Fig. 5:**
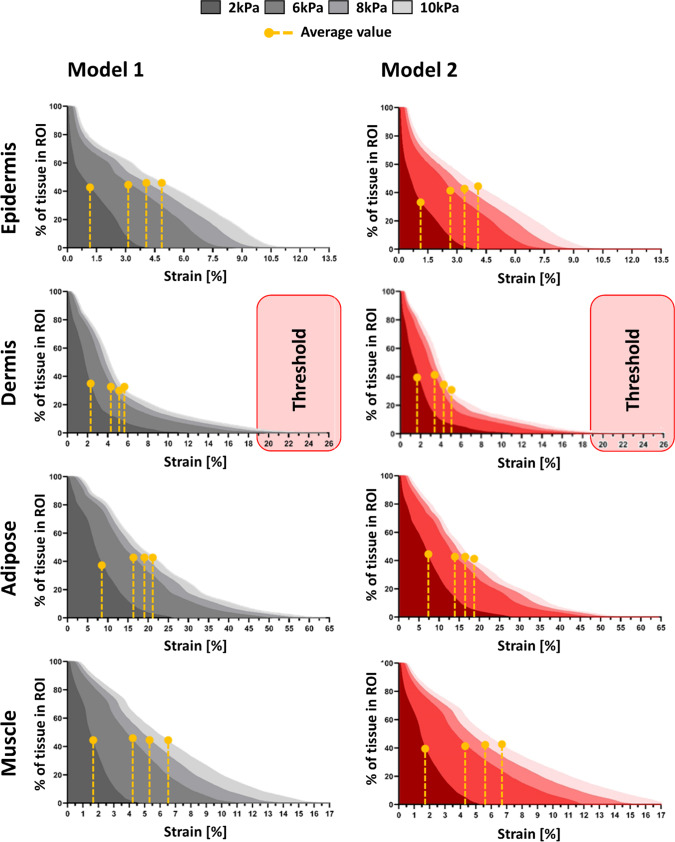
The volumetric soft tissue exposures to effective strains at the ROI of the two studied models (Model 1: left column, Model 2: right column). The tissue exposures to the relatively high strain values, defined here as strains above the 75th-percentile of the strain domain of the Model 1, were defined as the threshold for further analysis and compare the volume of tissue experiencing potentially damaging strain

To quantify the spatial extent of elevated mechanical exposure, stress and strain distributions were analyzed within a predefined 30 mm-diameter region of interest (ROI) beneath the electrode (Fig. 1). Cumulative distributions were computed at 2, 6, 8, and 10 kPa, and high-exposure thresholds were defined as the 75th percentile values obtained from Model 1 for each tissue and pressure level (Figs. 4, 5).

At 10 kPa, stress thresholds ranged from approximately 185–250 kPa in the epidermis and 8.5–11 kPa in the dermis, decreasing to 3–6 kPa in adipose and muscle. Corresponding strain thresholds were markedly higher in deeper tissues, reaching 48–65% in adipose and 13–17% in muscle, compared with 10–26% in epidermis and dermis. In addition, strain benchmarks related to therapeutic efficacy (3–6%) and potential cell damage (> 9%) were included for comparative interpretation (Marom et al. 2019; Slomka and Gefen 2012; Toume et al. 2017). Across all tissues, high stress remained spatially confined near the loading site, whereas high strain occupied a substantially larger tissue volume. Notably, Model 2 consistently exhibited a greater fraction of tissue exceeding both stress and strain thresholds, particularly at 8 and 10 kPa, indicating higher internal mechanical exposure under identical surface loading.

**Fig. 6:**
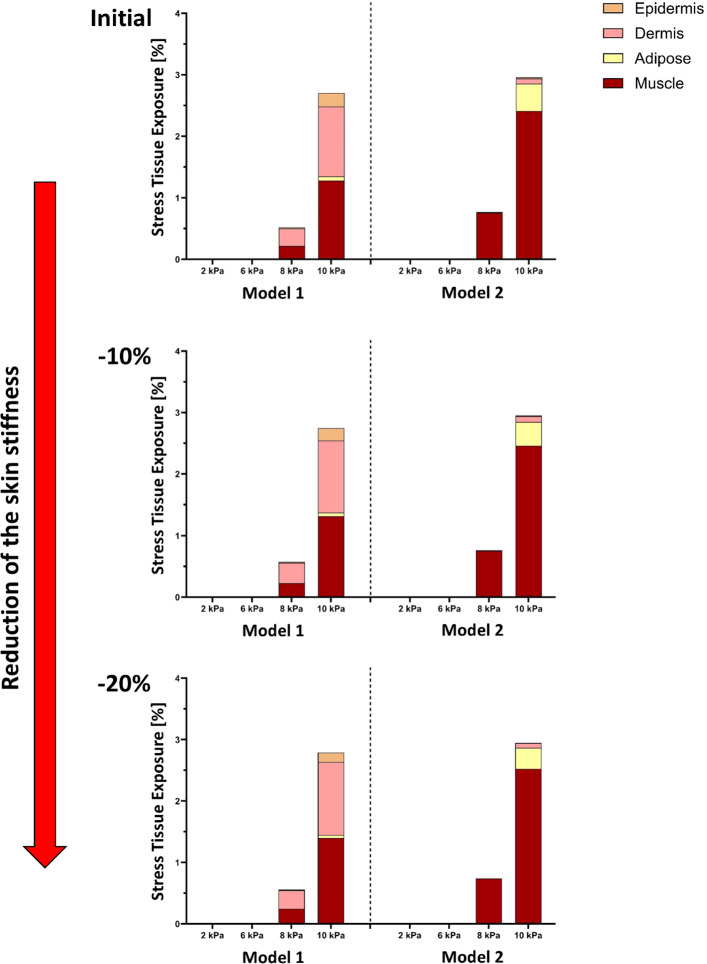
Tissue exposures at high stress levels under increasing mechanical loads for the two models, shown across three levels of skin stiffness (in top, middle, and bottom): initial (baseline), − 10%, and − 20% reduction. Tissue exposure is calculated as the percentage of total tissue volume experiencing stress above the defined threshold of that tissue. Each bar represents the cumulative exposure across the four modeled tissue layers (epidermis, dermis, adipose, muscle) under applied pressures of 2, 6, 8, or 10 kPa. As skin stiffness decreases (top to bottom), the percentage of tissue exposed to high stresses increases markedly, with muscle (Models 1 and 2) and dermis (Model 1) being the primary load-bearing layers

**Fig. 7:**
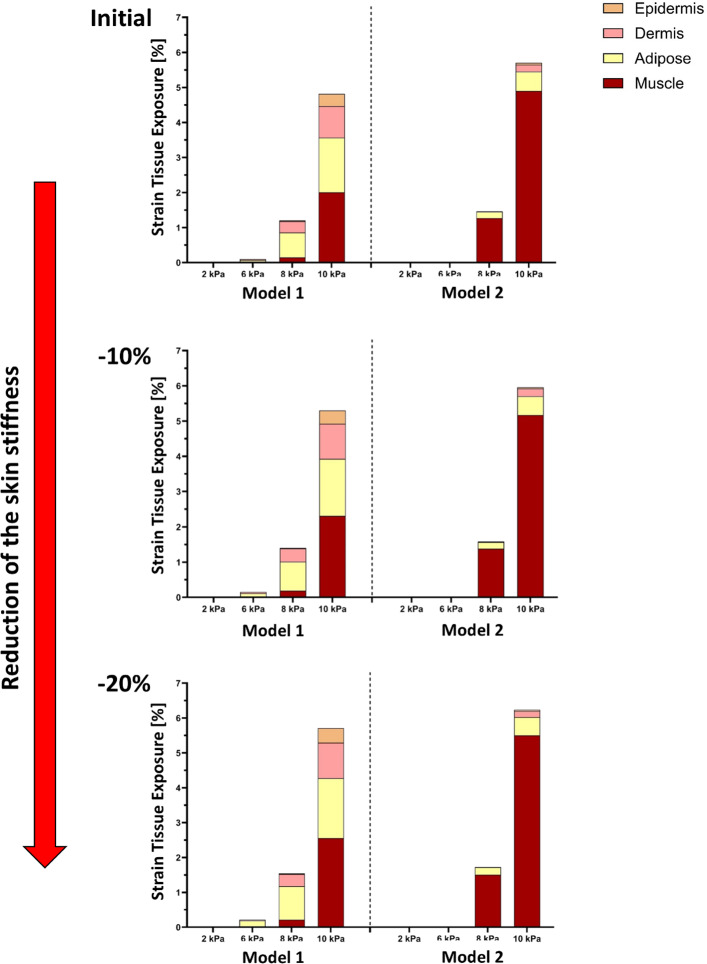
Tissue exposures at high strain levels under increasing mechanical loads for the two models, shown across three levels of skin stiffness (in top, middle, and bottom): Initial (baseline), − 10%, and − 20% reduction. Tissue exposure is calculated as the percentage of total tissue volume exceeding a defined strain threshold of that tissue. Each bar represents cumulative exposure across four tissue layers (epidermis, dermis, adipose, muscle) under applied pressures of 2, 6, 8, and 10 kPa. As skin stiffness decreases (top to bottom), a greater proportion of tissue volume is exposed to high strains, with muscle consistently being the most affected layer (Models 1 and 2). The dermis and adipose tissues also show increased exposure with softening (Model 1), indicating elevated vulnerability to deformation

Tissue exposure to elevated stress and strain was further evaluated across pressure magnitudes from 2 to 10 kPa under baseline stiffness and under uniform stiffness reductions of 10% and 20% (Figs. 6, 7). Across all stiffness conditions, appreciable high-stress and high-strain exposure occurred only at the highest pressure level (10 kPa), whereas exposure at 2–8 kPa remained negligible.

At baseline stiffness, Model 1 exhibited a distributed stress response at 10 kPa, with exposure shared between the dermis (1.3%) and muscle (1.4%, Fig. 6, upper panel). In contrast, Model 2 showed a markedly concentrated response, with stress exposure localized almost entirely in the muscle layer, reaching 2.7%. A similar distinction was observed for strain exposure (Fig. 7, upper panel): Model 1 displayed deformation across adipose, muscle, and dermis (2.0%, 1.3%, and 0.6%, respectively), whereas Model 2 was dominated by muscle strain alone (4.0%).

With a 10% reduction in stiffness, overall tissue exposure increased in both models, but redistribution patterns diverged. In Model 1, additional stress and strain remained shared between dermis and muscle (1.5–1.6%), indicating continued load distribution across layers. In Model 2, however, further softening amplified preferential load transfer to muscle, with stress exposure increasing to 2.9% and strain to 4.4%, while other layers contributed minimally (Figs. 6, 7, middle panels).

At the most compliant condition (20% stiffness reduction), total stress-exposed volume at 10 kPa increased to 3.8% in Model 1 and 4.3% in Model 2 (Fig. 6, lower panel). In Model 1, this increase remained distributed between muscle and dermis (1.8% each), whereas in Model 2, muscle accounted for nearly the entire stress exposure (3.2%), reflecting a pronounced shift toward deep-tissue load concentration. Strain exposure followed the same trend, rising to 6.0% in Model 1 and 7.1% in Model 2, with muscle consistently dominating in Model 2 (Fig. 7, lower panel).

**Fig. 8:**
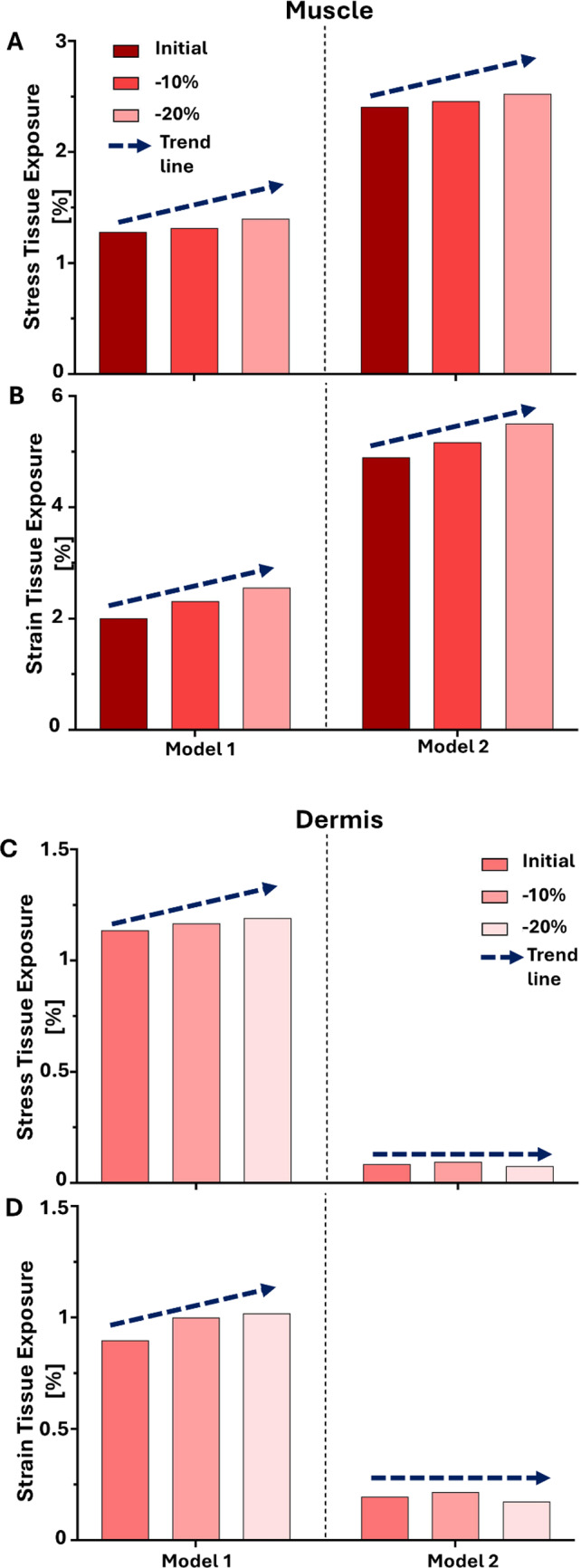
Muscle tissue exposures to high stress (**a**) and high strain (**b**) and dermal tissue exposures to high stress (**c**) and high strain (**d**) under 10 kPa loading in the two models, shown across three stiffness conditions: Initial (baseline), − 10%, and − 20% reduction. Exposure is expressed as the percentage of muscle/dermis volume exceeding the defined stress or strain threshold. Bars represent muscle/dermis exposure values under the applied pressure of 10 kPa. As stiffness decreases, exposure increases progressively in both models, confirming that muscle is the most vulnerable layer to elevated loading when tissue softening is present. In Model 1, exposures increase progressively with stiffness reduction, indicating heightened dermal vulnerability under tissue softening

Layer-specific analysis confirmed that muscle was the most mechanically vulnerable tissue across all stiffness conditions (Fig. 8). Muscle exposure increased progressively with softening in both models but remained consistently higher in Model 2. In contrast, dermal exposure increased with softening in Model 1 but remained negligible in Model 2, underscoring the role of superficial tissue composition in determining whether mechanical overload localizes in dermis or deep muscle. Across all stiffness conditions, tissue exposure at 2, 6, and 8 kPa remained minimal, indicating that substantial mechanical risk emerged primarily under high localized pressure.

## Discussion

This study used FE modeling to investigate how anatomical and mechanical differences in soft tissues influence tissue exposure to pressure-induced stress and strain, with relevance to pressure ulcer risk. Two distinct models, Models 1 and 2, were built based on published data for skin and underlying tissue properties, incorporating differences in tissue thickness, stiffness, and density to reflect anatomical variability (Blaak 2001; Feng et al. 2022; Hattori et al. 1991; Hoffmann et al. 2022; Karla et al. 2016; Lee and Hwang 2002; Levy et al. 2013; Liang and Boppart 2010; Nosslinger et al. 2022; Oltulu et al. 2018; Rabiatul et al. 2021; Sachs et al. 2021; Wang et al. 2023; Zhao et al. 2020). By simulating external pressures commonly associated with surface contact (e.g., from medical devices or immobility), the analysis enabled direct comparison of mechanical load transmission and strain propagation patterns across epidermal, dermal, adipose, and muscle layers (Fig. 1).

Our simulations showed that localized surface loading led to non-uniform stress and strain propagation through the soft tissue layers, with marked differences between Models 1 and 2. Although direct experimental validation was not performed, the predicted mechanical patterns are consistent with previously validated experimental and computational studies of pressure-induced tissue loading. Prior work has shown that localized surface compression leads to high stress concentrations in superficial layers, while large strains accumulate in deeper adipose and muscle tissues due to geometric confinement and tissue compliance (Linder-Ganz and Gefen 2004; Oomens et al. 2015; Stekelenburg et al. 2008). The agreement between the present results and these established biomechanical trends supports the physiological plausibility of the predicted stress and strain distributions. Material parameters were not calibrated to a specific experimental dataset but were selected to ensure internal mechanical consistency across tissue layers. All soft tissues were modeled using the same constitutive formulation with near-incompressible behavior and stiffness ranges consistent with previously published multilayer soft tissue models. Importantly, relative stiffness contrasts between layers were preserved to reflect physiological load-sharing mechanisms, enabling meaningful comparison of stress and strain responses across models. Layer-specific responses at 2 kPa (Figs. 2, 3) revealed distinct depth-dependent patterns of stress and strain between the two models. In both, epidermal stress was highest; however, in Model 1, it declined sharply with depth, whereas in Model 2, stress propagated more uniformly, suggesting reduced tissue cushioning by superficial tissues and greater transmission to deeper layers. Strain localized primarily in the adipose and muscle layers, even under this low pressure. Notably, Model 2 exhibited relatively higher strain in the muscle, indicating early mechanical vulnerability in deeper tissues linked to anatomical difference. At higher loading magnitudes (8–10 kPa), all models consistently exhibited greater internal stress and strain concentrations, particularly in the dermis, adipose, and muscle layers (Figs. 6, 7). These results align with previous reports on anatomical sites prone to pressure ulcer formation, where deeper tissues often experience elevated mechanical loads even when surface pressure remains moderate (Oomens et al. 2015). Importantly, cumulative stress and strain distributions demonstrated that a larger percentage of tissue in Model 2 exceeded the defined mechanical thresholds, which were based on the 75th percentile values from Model 1 (Figs. 4, 5). This suggests that individuals with certain anatomical and stiffness characteristics of adipose and muscle layers, may be more susceptible to internal tissue damage under similar loading conditions. The inclusion of both therapeutic (3–6%) and damaging (> 9%) strain thresholds allowed us to estimate deformation levels relevant to clinical outcomes such as impaired perfusion and cell death (Marom et al. 2019; Slomka and Gefen 2012; Toume et al. 2017).

In order to simulate aging effects or pathological softening the soft tissue stiffness was reduced to 10% and 20%, both models exhibited increased exposure to damaging mechanical states. However, the effect was more evident in Model 1, where softening led to a broader distribution of high strain in the dermis, adipose, and muscle. In contrast, Model 2 exhibited consistently high values primarily in the muscle layer, even at baseline stiffness, indicating a heightened risk of deep tissue injury that may develop before visible damage appears on the skin surface, highlighting the clinical relevance of early detection and targeted preventative care (Figs. 6, 7). These findings demonstrate that variations in tissue composition substantially influence both the magnitude and localization of mechanical risk. Layer-specific analysis further emphasized these differences (Fig. 8). Muscle consistently emerged as the most vulnerable tissue in both models, with exposures increasing progressively as stiffness was reduced. This pattern was particularly pronounced in Model 2, where muscle loading dominated even at baseline stiffness. By contrast, dermal vulnerability increased with softening in Model 1 but remained negligible in Model 2, highlighting that superficial cushioning strongly influences whether dermis or muscle becomes the primary site of overload.

The choice to apply clinically relevant and consistent loads enabled standardized comparisons across models. However, it is important to note that both models were developed using averaged anatomical and mechanical parameters (Blaak 2001; Hattori et al. 1991; Oltulu et al. 2018). While this supports controlled comparisons, it also limits direct generalizability to individuals, as it does not capture the full spectrum of inter-individual variability in body composition, tissue quality, or anatomical proportions. The use of average anatomical configurations was deliberate, enabling isolation of the biomechanical effects of tissue thickness and stiffness under identical loading and boundary conditions. Incorporating subject-specific geometry, while essential for individualized clinical diagnosis, introduces multiple confounding variables that can obscure the mechanical contribution of tissue composition itself. The present work therefore serves as a foundational biomechanical analysis, identifying tissue layers and mechanical metrics most sensitive to anatomical variability and providing a rational basis for future patient-specific and multiscale modeling efforts.

Modeling simplifications and limitations should be considered when interpreting the results. Soft tissues were represented using an isotropic Neo-Hookean constitutive law with viscoelastic relaxation, which does not fully capture nonlinear, anisotropic, or rate-dependent behavior. Sensitivity analysis was limited to uniform 10% and 20% stiffness reductions representing moderate physiological and pathological softening; however, relative trends, particularly redistribution of strain toward deeper tissues and differential vulnerability between models, were robust across this range. The geometric domain was simplified to a layered planar block with symmetry conditions, and loading was applied as static uniaxial compression, neglecting shear, friction, and dynamic effects associated with patient movement or device interaction. Biological processes such as perfusion, ischemia, inflammation, and damage accumulation were not modeled, nor was direct experimental validation performed. Accordingly, stress and strain outputs are interpreted as indicators of mechanical exposure rather than predictors of tissue injury.

Within this scope, the present study adopts a controlled comparative approach rather than a patient-specific or fully mechanobiological framework. Stress and strain metrics are interpreted as mechanobiology-informed exposure indicators, contextualized using experimentally observed cellular response ranges rather than explicit damage modeling. Experimental evidence links elevated tissue deformation to microvascular collapse, impaired perfusion, and membrane disruption, recognized precursors to pressure injury, supporting the relevance of the mechanical patterns identified here. While the study does not propose new bedside measurements or replace established risk-assessment tools such as the Braden Scale, it offers a biomechanical framework that complements clinical scoring by explaining inter-individual differences in internal mechanical vulnerability. The present findings provide mechanistic insight into how anatomical variability influences internal mechanical exposure under localized pressure, yet they do not yet support direct implementation of personalized prevention strategies. Translation to clinical practice will require patient-specific imaging, experimental validation, integration with clinical workflows, and prospective evaluation. The current work therefore represents a foundational step toward such applications rather than a deployable clinical solution. Future extensions will involve coupling predicted stress–strain fields to models of perfusion, ischemia duration, and cellular mechanotransduction to simulate injury initiation and progression.

From a clinical perspective, the present results provide mechanistic insight into why pressure-injury risk may vary substantially between individuals exposed to similar external pressures. The simulations presented here show that anatomical differences in tissue thickness and stiffness can substantially change internal stress and strain distributions, leading to elevated deep-tissue deformation even when superficial pressure appears moderate. Specifically, elevated strain levels in muscle demonstrate a potential mechanism for the development of deep tissue injury that precedes visible skin damage. While the present study does not predict injury or prescribe interventions, these results indicate that increased clinical vigilance may be warranted for individuals with reduced soft tissue cushioning or tissue softening and emphasize the importance of considering internal mechanical exposure, rather than surface pressure alone, when interpreting pressure-related risk.

## Data Availability

Data available from corresponding author upon reasonable request.

## Abbreviations

AUC: Area under the curve
FEM: Finite element modeling
PUs: Pressure ulcers
ROI: Region of interest
